# Predicting Prolonged Length of Stay in Acute Pancreatitis: Comparison of the CRP-to-Albumin Ratio with Other Inflammatory and Immunoutritional Indices

**DOI:** 10.3390/metabo16050320

**Published:** 2026-05-11

**Authors:** Ümit Karatepe, Berçem Afşar Karatepe

**Affiliations:** 1 Department of Anesthesiology and Reanimation, Elazığ Fethi Sekin City Hospital, Elazığ 23300, Turkey; 2 Department of Internal Medicine, Elazığ Fethi Sekin City Hospital, Elazığ 23300, Turkey; b.afsarkaratepe@saglik.gov.tr

**Keywords:** acute pancreatitis, C-reactive protein/albumin ratio, length of stay, negative predictive value, immuno-nutritional ratios

## Abstract

**Objective**: Due to the varied clinical manifestations of acute pancreatitis (AP), prompt identification of patients predisposed to extended hospitalization is essential for efficient resource allocation. This study assessed the predictive efficacy of inflammatory and immunonutritional ratios—namely, C-reactive protein/albumin ratio (CAR), neutrophil-to-lymphocyte ratio (NLR), platelet-to-lymphocyte ratio (PLR), and hemoglobin, albumin, lymphocyte, and platelet score (HALP)—in predicting hospitalizations lasting more than 7 days. **Methods:** A retrospective cohort analysis was performed on 306 patients treated at a tertiary center from June 2020 to June 2025. We used Mann–Whitney U tests, ROC analysis, and multivariate logistic regression models to evaluate the relationship between admission laboratory-derived ratios and length of stay. **Results:** In total, 27.5% (n = 84) of the cohort experienced prolonged hospitalization. Individual markers exhibited moderate discrimination; however, procalcitonin and CAR displayed high negative predictive values (>85%), demonstrating clinical utility in excluding prolonged hospital stays. Multivariate analysis revealed advanced age (*p* < 0.001) and increased CAR (*p* < 0.001) as the most significant independent predictors. On the other hand, the HALP score was much lower in the group that stayed longer, but it was not an independent predictor in the multivariate model. **Conclusions:** Older age and a higher CAR are both independent factors that can predict longer hospital stays in AP. The high negative predictive value of CAR is important because it represents a reliable way to exclude prolonged hospitalization. Low CAR levels at admission may help clinicians identify patients eligible for early discharge, thereby optimizing bed management.

## 1. Introduction

Acute pancreatitis (AP) is a common gastrointestinal emergency with a variable clinical course. According to the revised Atlanta classification, it may present as a mild, self-limiting disease or as a more severe condition associated with local complications, persistent organ failure, and considerable morbidity [1]. Although many patients recover with supportive care, some require longer hospitalization because of persistent symptoms, delayed recovery, ongoing inflammation, or increased supportive care needs. Identifying patients at risk for prolonged hospital stay at an early stage may therefore be useful in daily practice, particularly for monitoring, resource allocation, and discharge planning [2,3].

The course of AP is influenced not only by the extent of local pancreatic injury but also by the severity of the systemic inflammatory response and the patient’s physiological reserve [3,4]. Experimental and mechanistic studies have shown that sustained intracellular calcium overload, mitochondrial dysfunction, and adenosine triphosphate (ATP) depletion contribute to acinar cell injury and intensify inflammatory pathways in AP [3,4,5]. In clinical practice, laboratory-based inflammatory and immunonutritional markers are better regarded as indirect indicators of systemic stress, inflammatory burden, and host reserve rather than as direct drivers of disease progression [3,4].

Most previous studies on laboratory-based prognostic markers in AP have focused on disease severity, organ failure, complications, or mortality. In comparison, fewer studies have specifically investigated predictors of hospital length of stay (LOS), even though LOS is a clinically relevant and resource-sensitive outcome [6,7,8]. Established scoring systems such as the Acute Physiology and Chronic Health Evaluation II (APACHE II), Bedside Index for Severity in Acute Pancreatitis (BISAP), Ranson score, and Computed Tomography Severity Index (CTSI) are still useful for risk assessment. However, delayed completion, relative complexity, or limited practicality at the bedside may restrict their routine use [7,8,9].

Among readily available admission markers, the neutrophil-to-lymphocyte ratio (NLR) and platelet-to-lymphocyte ratio (PLR) reflect different aspects of the systemic inflammatory response. The C-reactive protein-to-albumin ratio (CAR) combines inflammatory burden with nutritional and physiological status [9,10,11,12,13]. Procalcitonin has also been linked to severe inflammation and infected pancreatic necrosis in AP [14,15]. The hemoglobin, albumin, lymphocyte, and platelet (HALP) score reflects a broader immunonutritional profile, although evidence regarding its role in AP is still limited [16]. The relationship between these admission markers and prolonged hospital stay in AP, however, has not been clearly defined. Therefore, this study aimed to compare selected admission inflammatory and immunonutritional markers as predictors of prolonged hospital stays for patients hospitalized with AP.

## 2. Materials and Methods

This study has a single-center, retrospective cohort design. Approval was obtained from the Elazığ Fethi Sekin City Hospital Non-Interventional Research Ethics Committee (Date: 4 September 2025; Decision No.: 2025/14-09); this study was conducted in accordance with the Declaration of Helsinki.

This study was conducted by retrospectively examining the medical records of patients who presented to our hospital’s emergency department between June 2020 and June 2025, were diagnosed with acute pancreatitis according to the revised Atlanta classification, and were admitted to internal medicine, general surgery clinics, or the intensive care unit.

In the diagnostic process, at least two of the following criteria were used:

(i) Typical epigastric pain with acute onset, band-like distribution, and radiation to the back;
(ii) Serum amylase or lipase levels at least three times higher than the upper limit of normal (>3x);
(iii) The presence of characteristic radiological findings of pancreatic inflammation on abdominal ultrasonography (USG) or computed tomography (CT).

Inclusion criteria: must be 18 years of age or older and diagnosed with acute pancreatitis according to the revised Atlanta classification.

Exclusion criteria include a history of chronic pancreatitis, pancreatic cancer, or other solid or hematological malignancies; pregnancy; chronic inflammatory or autoimmune diseases; bone marrow disorders; the use of immunosuppressive drugs; and anemia. After application of these criteria, 306 patients were included in the final analysis.

**Figure 1:**
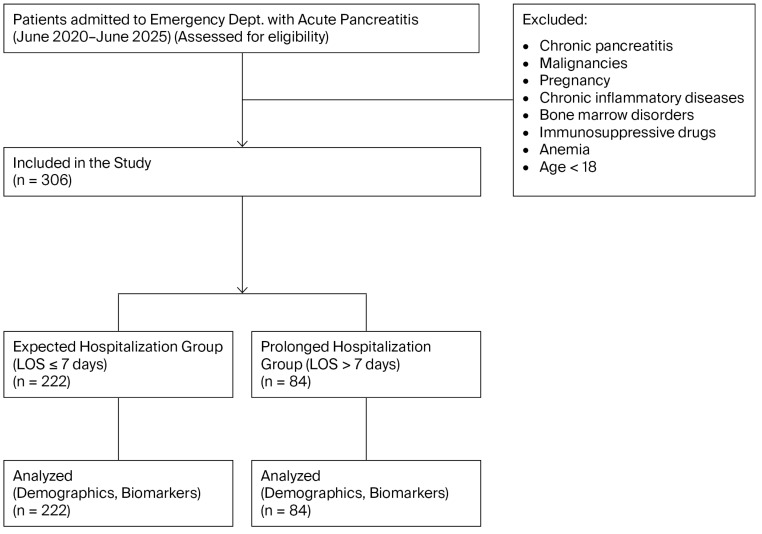
Flowchart of patient selection, exclusion process, and final study inclusion.

Demographic data (age and sex), clinical variables (mode of admission, length of stay [LOS], and intensive care unit requirement), and laboratory results were obtained from patient files and electronic medical records. The inflammatory and immunonutritional markers included in the analysis were based on the first blood samples obtained at initial presentation to the emergency department. All measurements were performed in the hospital’s central laboratory using routinely calibrated automated analyzers according to standard procedures. NLR, PLR, HALP, and the C-reactive protein-to-albumin ratio (CAR) were calculated from these admission values. Patient selection and study inclusion are summarized in Figure 1.

The primary endpoint was defined as a prolonged hospital stay (LOS > 7 days). LOS ≤ 7 days was classified as the “expected admission” group, while LOS > 7 days was classified as the “prolonged admission” group.

### Statistical Analysis

The Statistical Package for Social Sciences (SPSS) version 22.0 was used for statistical analyses. The categorical variables are presented as frequencies and percentages. The continuous variables were assessed using the Kolmogorov–Smirnov test and histograms to find out if their distributions were normal or not. The normally distributed numerical parameters were compared using Student’s *t*-test in groups, while those with non-normal distributions were analyzed with Mann–Whitney U tests. The categorical variables were compared using chi-squared or Fisher’s exact tests where appropriate. The accuracy of clinical and laboratory parameters to predict length of stay was evaluated with receiver operating characteristic (ROC) analysis. The accuracy of tests was measured using the area under the ROC curve. An area under the curve (AUC) close to 1 represents a perfect diagnostic test, whereas an area of 0.5 represents a worthless test. A statistical difference was considered when the *p*-value < 0.05. The parameters that were identified and were significantly different between expected and prolonged hospitalization in univariate analyses were put into the equation for logistic regression analysis. Variables eligible for inclusion in the multivariate analysis were tested for collinearity. Additionally, multivariable logistic regression analysis with a stepwise backward approach was performed. Variables that remained significant (*p* < 0.05) in the multivariate model were considered as independent predictors for prolonged hospitalization. Hosmer–Lemeshow goodness of fit statistics were used to assess model fit. Odds ratios (ORs) and 95% confidence intervals (CIs) were calculated for each predictor.

## 3. Results

A total of 306 inpatients were included: 222 (72.5%) had an expected length of stay (LOS ≤ 7 days), and 84 (27.5%) experienced prolonged hospitalization (LOS > 7 days). Patients with prolonged LOS were older (median 71 (33–93) vs. 63 (20–96) years; *p* < 0.001), while sex distribution did not differ between groups (male 33.3% vs. 34.2%; *p* = 0.882). Hemoglobin, platelets, and albumin were comparable between groups (all *p* > 0.05). In contrast, inflammatory and hematologic indices showed marked differences: prolonged LOS was associated with higher neutrophil counts (median 9090 vs. 7520/µL; *p* < 0.001), lower lymphocyte counts (median 1090 vs. 1350/µL; *p* = 0.004), higher CRP (median 37.0 vs. 12.3 mg/L; *p* < 0.001), higher procalcitonin (median 1.15 vs. 0.25 ng/mL; *p* < 0.001), higher NLR (9.16 vs. 5.55; *p* < 0.001), higher PLR (235.35 vs. 171.50; *p* < 0.001), lower HALP (22.35 vs. 28.30; *p* = 0.001), and higher CRP/albumin ratio (1.01 vs. 0.33; *p* < 0.001) (Table 1).

**Figure 2:**
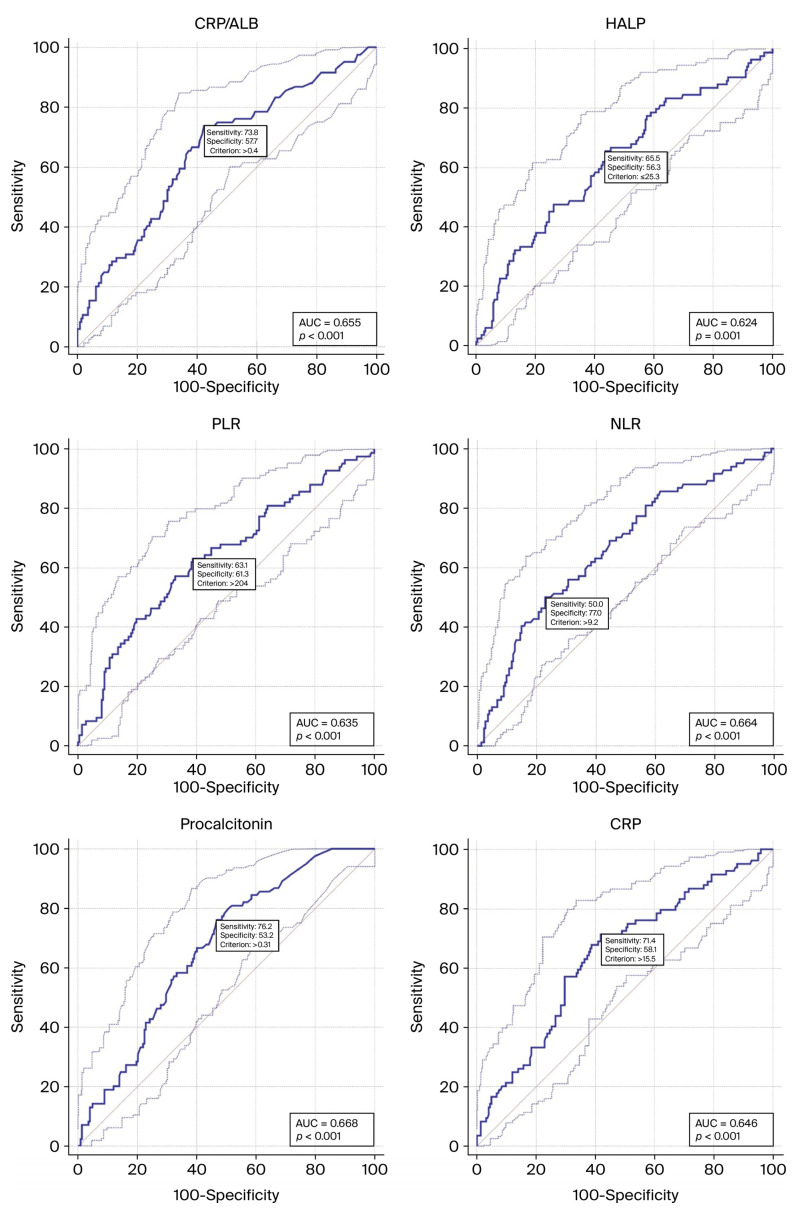

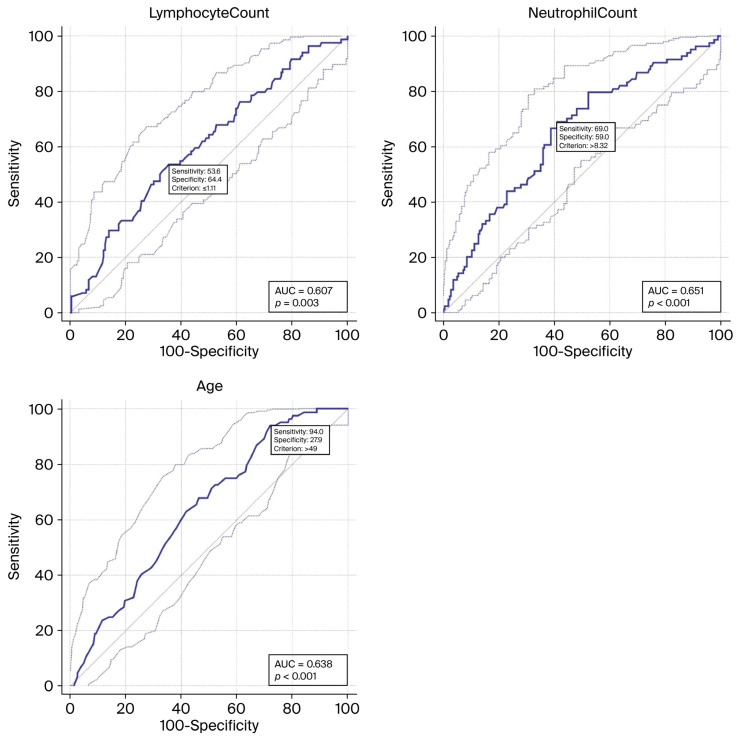
ROC curve analyses regarding Table 2.

In the ROC analysis, individual markers showed modest discrimination for prolonged LOS. The highest AUCs were observed for procalcitonin (AUC 0.668; cutoff 0.31 ng/mL; sensitivity 76.2%; specificity 53.2%), NLR (AUC 0.664; cutoff 9.2; sensitivity 50.0%; specificity 77.0%), and CRP/albumin (AUC 0.655; cutoff 0.40; sensitivity 73.8%; specificity 57.7%). Neutrophil count (AUC 0.651; cutoff 8320/µL; sensitivity 69.0%; specificity 59.0%) and CRP (AUC 0.646; cutoff 15.5 mg/L; sensitivity 71.4%; specificity 58.1%) performed similarly. Age yielded an AUC of 0.638 (cutoff 49 years; sensitivity 94.0%; specificity 27.9%), PLR an AUC of 0.635 (cutoff 204; sensitivity 63.1%; specificity 61.3%), HALP an AUC of 0.624 (cutoff 25.3; sensitivity 65.5%; specificity 56.3%), and lymphocyte count an AUC of 0.607 (cutoff 1110/µL; sensitivity 53.6%; specificity 64.4%) (all *p* ≤ 0.003). Notably, several markers provided relatively high negative predictive values (NPVs), including procalcitonin (85.5%), CRP/albumin (85.3%), CRP (84.3%), NLR (80.3%), and PLR (81.4%), indicating utility in ruling out prolonged LOS at the specified thresholds (Table 2). Figure 2 displays the ROC curves for these indicators.

In univariable logistic regression, older age (OR 1.032 per year; 95% CI 1.015–1.048; *p* < 0.001), higher NLR (OR 1.034 per unit; 95% CI 1.013–1.056; *p* = 0.001), higher PLR (OR 1.001 per unit; 95% CI 1.0001–1.002; *p* = 0.011), lower HALP (OR 0.987 per unit; 95% CI 0.975–0.999; *p* = 0.037), and higher CRP/albumin (OR 1.244 per unit; 95% CI 1.115–1.388; *p* < 0.001) were associated with prolonged LOS, whereas procalcitonin was not significant in the unadjusted model (OR 1.016; 95% CI 0.990–1.042; *p* = 0.244). In the multivariable model, age (adjusted OR 1.029 per year; 95% CI 1.012–1.046; *p* = 0.001) and CRP/albumin (adjusted OR 1.208 per unit; 95% CI 1.077–1.354; *p* = 0.001) remained independent predictors of prolonged hospitalization. NLR showed a borderline association after adjustment (adjusted OR 1.022; 95% CI 0.999–1.044; *p* = 0.059), while PLR, HALP, and procalcitonin did not retain significance in the adjusted analysis (Table 3).

Overall, prolonged LOS was characterized by older age and a pro-inflammatory laboratory profile (higher neutrophil count, CRP, procalcitonin, NLR, and PLR; lower lymphocyte count and HALP), with the CRP/albumin ratio and age emerging as independent predictors in multivariable analysis.

## 4. Discussion

In our cohort, several admission inflammatory and immunonutritional markers were associated with prolonged hospital stays in patients with AP. After adjustment, however, only older age and CAR remained independently associated with prolonged hospital stays. This is clinically significant, as the duration of stay in acute pancreatitis (AP) indicates not only the initial presentation but also the subsequent recovery trajectory and the necessity for continued care. The CAR cutoff of 0.4 was derived from ROC analysis in our cohort. Differences in reported cutoff values across studies may be related to variations in patient populations, clinical outcomes, and timing of measurement. Recent studies have also demonstrated a correlation between prolonged hospitalization and both clinical features and laboratory findings. Koçkan et al. identified predictors of longer stays in patients with mild-to-moderate AP, whereas Obaitan et al. reported several factors associated with increased length of stay in acute necrotizing pancreatitis [17,18]. Wang et al. also found that blood glucose levels at admission were associated with hospital stay duration [19]. More recently, Algin et al. showed that CAR and other CRP- and albumin-based indices were independently associated with prolonged hospital stays [20].

Routine laboratory tests obtained at admission derive these markers, eliminating the need for additional procedures or complex calculations. Since they are available at the time of initial evaluation, they may help support early clinical assessment of disease course and hospitalization burden. Their simplicity may also support their use in routine clinical assessment. From a biological standpoint, the correlation between inflammatory markers and extended hospitalizations is justifiable. AP is characterized by a systemic inflammatory response that extends beyond local pancreatic injury and may complicate the clinical course in some patients [21].

Among the evaluated markers, CAR emerged as the strongest predictor of prolonged hospitalization. Because it reflects both inflammatory activity and physiological reserve, it may provide broader clinical information than isolated inflammatory indices alone. This may explain why it remained independently associated with a longer hospital stay after adjustment for age and other variables. Recent studies have likewise suggested that CRP- and albumin-based composite indices, including CAR, may help identify patients at risk for prolonged hospitalization in AP [20]. Table 4 summarizes the threshold identified in the present study together with the corresponding results for the evaluated markers. Although this value should not be interpreted as a universal clinical cutoff, presenting it may still help in interpreting our findings in a more practical way.

In the univariable analyses, NLR and PLR were also associated with longer hospital stays. This is in line with earlier studies linking these indices to severe AP, pancreatic necrosis, and adverse clinical outcomes [22,23,24,25]. Their loss of significance in the multivariable model suggests that they may reflect the overall inflammatory response rather than independently determine the length of hospitalization. Procalcitonin may be interpreted in a similar way, as previous studies have linked it more closely to severe inflammation and infected pancreatic necrosis in AP [26,27].

Patients with longer hospitalization also had lower HALP scores, suggesting that poorer immunonutritional status may be associated with delayed recovery. However, HALP was not independently associated with prolonged stay in the adjusted model. Given the limited evidence regarding HALP in AP, this finding should be interpreted with caution and considered exploratory [28,29,30].

**Table 4:** The predictive performance of parameters in AP according to key studies in the literature.

Ratio and Score	Clinical Significance	Critical Cutoff Values (Mean)	Reference
NLR	Predicts severe pancreatitis (SAP) and the need for intensive care (ICU) within the first 24 h.	>4.7–10.6	Fonseca, T. et al. [22] Azab et al. [23]
PLR	Associated with the development of necrosis and mortality.	>150–200	Yang et al. [14]
HALP	A low score indicates poor prognosis and risk of organ failure.	Variable (low values are risky)	Xu et al. [29]
CAR	Superior to CRP in predicting mortality and complications.	>0.5–2.0	Kaplan et al. [12]
PCT	Gold standard for diagnosing infected pancreatic necrosis.	>0.5 ng/mL (Sepsis risk) >2.0 ng/mL (high risk)	Mofidi et al. [16]

The 7-day threshold used to define prolonged hospitalization should also be interpreted in light of the study design. Since there is no universally accepted definition of prolonged stay in AP, we selected this cutoff because it corresponded to the median length of stay in our cohort and allowed a practical distinction between shorter and longer hospitalizations. Evidence-based reviews have shown that patients with mild AP are often discharged within the first week, which supports the clinical relevance of this threshold [30]. Nevertheless, it should be regarded as an operational definition for this study rather than a universal clinical standard.

In general, a number of inflammatory and immunonutritional markers at admission were linked to longer hospital stays in AP. However, only older age and CAR stayed linked after adjustments were made. These findings suggest that CAR may be a useful marker for identifying patients who are more likely to require a longer hospital stay.

## 5. Limitations

This study presents several issues. First, the retrospective single-center approach limits causal inference and may impede the generalizability of the findings. Second, this study focused on routinely accessible admission-based markers, but critical clinical variables such as acute pancreatitis severity classifications, comorbidity burden, specific etiological subgroups, and treatment-related factors were not uniformly available for all patients and therefore could not be incorporated into the adjusted model. Third, all inflammatory and immunonutritional markers were derived from the first blood samples acquired at presentation, and variations in these parameters during hospitalization were not evaluated. As mentioned in the previous section, the definition of prolonged hospitalization as a length of stay exceeding seven days should be considered an operational criterion for studies rather than a universal clinical standard. Ultimately, despite numerous markers demonstrating significant associations in univariable studies, it is crucial to validate these findings in larger, more heterogeneous populations prior to their application in clinical practice.

## 6. Conclusions

Our findings suggest that CAR may have potential value as an admission-based marker for the early assessment of prolonged hospitalization in acute pancreatitis. However, multicenter prospective studies incorporating disease severity and serial measurements are required to confirm this observation.

## Figures and Tables

**Table 1:** Demographic and laboratory characteristics of the patients.

	Total (n = 306)	Expected Hospitalization (7 Days and Under) (n = 222)	Prolonged Hospitalization (Above 7 Days) (n = 84)	*p*
Age, median (min–max) years	66 (20–96)	63 (20–96)	71 (33–93)	**<** **0.001**
Sex				
Male	104 (34)	76 (34.2)	28 (33.3)	0.882
Female	202 (66)	146 (65.8)	56 (66.7)	
Hb, g/dL (mean ± SD)	13.7 ± 1.8	13.6 ± 1.7	13.8 ± 2	0.434
Plt, median (min–max) 10^9^/L	245 (53–534)	239.5 (75–449)	251 (53–534)	0.156
Neutrophil, median (min–max) %	8265 (1150–36,980)	7520 (1150–35,220)	9090 (1260–36,980)	**<0.001**
Lymphocyte, median (min–max) %	1275 (80–10,710)	1350 (80–8700)	1090 (100–10,710)	**0.004**
Albumin, median (min–max) g/L	37 (22–50)	37 (24–50)	37 (22–49)	0.180
CRP, median (min–max) mg/L	15.9 (0.4–460)	12.3 (0.4–408)	37 (1.84–460)	**<0.001**
Procalcitonin, median (min–max) ng/mL	0.46 (0.001–99)	0.25 (0.001–99)	1.15 (0.04–42.7)	**<0.001**
NLR, median (min–max)	6 (0.54–68.7)	5.55 (0.54–68.7)	9.16 (0.72–58)	**<0.001**
PLR, median (min–max)	189.4 (27.2–2700)	171.5 (34.4–2357)	235.35 (27.2–2700)	**<0.001**
HALP, median (min–max)	25.6 (1.46–241)	28.3 (1.88–145)	22.35 (1.46–241)	**0.001**
CRP/ALB, median (min–max)	0.42 (0.01–13.9)	0.33 (0.01–9.48)	1.01 (0.04–13.9)	**<0.001**

**Table 2:** Results of receiver operating characteristic analysis to differentiate between expected and prolonged hospitalization.

Parameters	AUC (95% CI)	Cutoff	Sensitivity	Specificity	Positive Predictive Value (%)	Negative Predictive Value (%)	*p*
Age years	0.638	49	94	27.9	33.1	92.5	**<** **0.001**
Neutrophil %	0.651	8320	69	59	38.9	83.4	**<0.001**
Lymphocyte %	0.607	1110	53.6	64.4	36.3	78.6	**0.003**
CRP mg/L	0.646	15.5	71.4	58.1	39.2	84.3	**<0.001**
Procalcitonin ng/mL	0.668	0.31	76.2	53.2	38.1	85.5	**<0.001**
NLR	0.664	9.2	50	77	45.2	80.3	**<0.001**
PLR	0.635	204	63.1	61.3	38.1	81.4	**<0.001**
HALP	0.624	25.3	65.5	56.3	36.2	81.2	**0.001**
CRP/ALB	0.655	0.4	73.8	57.7	39.7	85.3	**<0.001**

**Table 3:** Univariable and multivariable logistic regression analysis of factors associated with prolonged hospitalization.

	Unadjusted		Adjusted	
Risk Factors	OR (95% CI)	*p*	OR (95% CI)	*p*
Age years	1.032 (1.015–1.048)	**<** **0.001**	1.029 (1.012–1.046)	**0.001**
Procalcitonin ng/mL	1.016 (0.990–1.042)	0.244		
NLR	1.034 (1.013–1.056)	**0.001**	1.022 (0.999–1.044)	0.059
PLR	1.001 (1.0001–1.002)	**0.011**		
HALP	0.987 (0.975–0.999)	**0.037**		
CRP/ALB	1.244 (1.115–1.388)	**<0.001**	1.208 (1.077–1.354)	**0.001**

## Data Availability

The original contributions presented in this study are included in the article. Further inquiries can be directed to the corresponding author.

## Abbreviations

The following abbreviations are used in this manuscript:

AP	Acute Pancreatitis
LOS	Length of Stay
SIRS	Systemic Inflammatory Response Syndrome
ICU	Intensive Care Unit
PCT	Procalcitonin
NLR	Neutrophil/Lymphocyte Ratio
PLR	Platelet/Lymphocyte Ratio
CRP	C-Reactive Protein
CAR	CRP/Albumin Ratio
HALP Score	Hemoglobin, Albumin, Lymphocyte, and Platelet Score
APACHE II	Acute Physiology and Chronic Health Evaluation II
BISAP	Bedside Index for Severity in Acute Pancreatitis
